# In silico drug discovery of SIRT2 inhibitors from natural source as anticancer agents

**DOI:** 10.1038/s41598-023-28226-7

**Published:** 2023-02-07

**Authors:** Mahmoud A. A. Ibrahim, Khlood A. A. Abdeljawaad, Eslam Roshdy, Dina E. M. Mohamed, Taha F. S. Ali, Gamal A. Gabr, Laila A. Jaragh-Alhadad, Gamal A. H. Mekhemer, Ahmed M. Shawky, Peter A. Sidhom, Alaa H. M. Abdelrahman

**Affiliations:** 1 Computational Chemistry Laboratory, Chemistry Department, Faculty of Science, Minia University, Minia, 61519 Egypt; 2 Medicinal Chemistry Department, Faculty of Pharmacy, Minia University, Minia, 61519 Egypt; 3 Department of Chemistry, Graduate School of Advanced Science and Engineering, Hiroshima University, Higashi-Hiroshima, Hiroshima 739-8526 Japan; 4 Department of Pharmacology and Toxicology, College of Pharmacy, Prince Sattam Bin Abdulaziz University, Al-Kharj, 11942 Saudi Arabia; 5 Agricultural Genetic Engineering Research Institute (AGERI), Agricultural Research Center, Giza, Egypt; 6 Department of Chemistry, Faculty of Science, Kuwait University, Safat, 13060 Kuwait; 7 Science and Technology Unit (STU), Umm Al-Qura University, Makkah, 21955 Saudi Arabia; 8 Department of Pharmaceutical Chemistry, Faculty of Pharmacy, Tanta University, Tanta, 31527 Egypt

**Keywords:** Cancer, Computational biology and bioinformatics

## Abstract

Sirtuin 2 (SIRT2) is a member of the sirtuin protein family, which includes lysine deacylases that are NAD^+^-dependent and organize several biological processes. Different forms of cancer have been associated with dysregulation of SIRT2 activity. Hence, identifying potent inhibitors for SIRT2 has piqued considerable attention in the drug discovery community. In the current study, the Natural Products Atlas (NPAtlas) database was mined to hunt potential SIRT2 inhibitors utilizing in silico techniques. Initially, the performance of the employed docking protocol to anticipate ligand-SIRT2 binding mode was assessed according to the accessible experimental data. Based on the predicted docking scores, the most promising NPAtlas molecules were selected and submitted to molecular dynamics (MD) simulations, followed by binding energy computations. Based on the MM-GBSA binding energy estimations over a 200 ns MD course, three NPAtlas compounds, namely NPA009578, NPA006805, and NPA001884, were identified with better Δ*G*_binding_ towards SIRT2 protein than the native ligand (SirReal2) with values of − 59.9, − 57.4, − 53.5, and − 49.7 kcal/mol, respectively. On the basis of structural and energetic assessments, the identified NPAtlas compounds were confirmed to be steady over a 200 ns MD course. The drug-likeness and pharmacokinetic characteristics of the identified NPAtlas molecules were anticipated, and robust bioavailability was predicted. Conclusively, the current results propose potent inhibitors for SIRT2 deserving more in vitro/in vivo investigation.

## Introduction

Sirtuins are superior histone deacetylases class III (HDAC III), which are NAD^+^-dependent protein deacetylases^1,2^. Recent studies have proven that sirtuins not only deacetylate but also catalyze many post-translational modulations involving demyristoylation and desuccinylation^3–5^. The mammalian genome encodes seven different members (SIRT1-7), which vary in their subcellular localization^6,7^. SIRT6 and SIRT7 are centered on the nucleus, SIRT3-5 are caged in mitochondria, while SIRT1 and SIRT2 house both cytoplasm and nucleus^8^.

**Figure 1:**
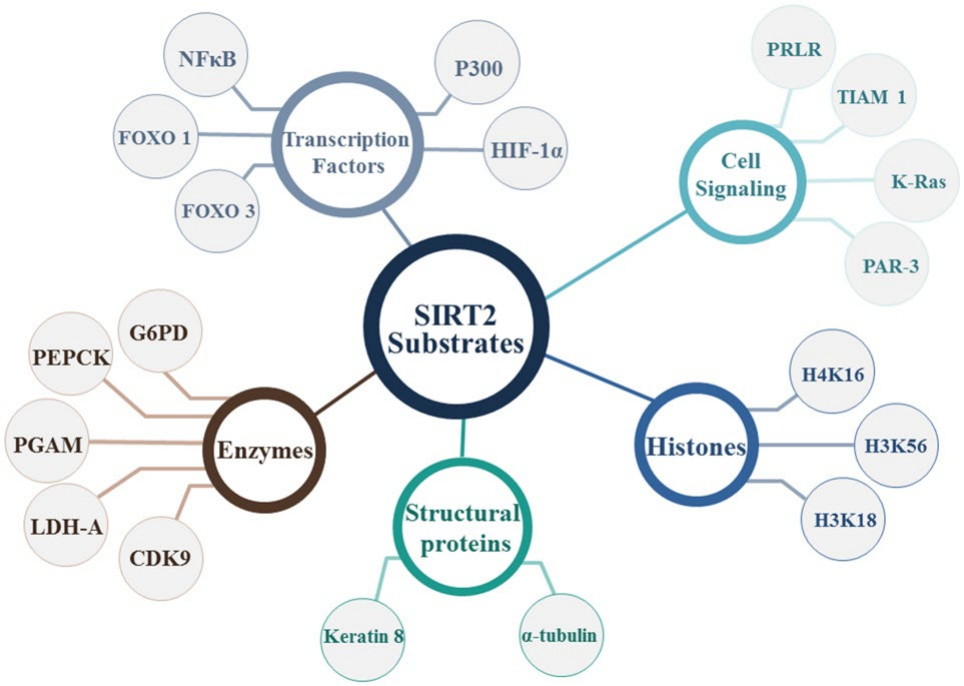
Illustrative diagram for SIRT2 substrates.

SIRT2 substrates, as shown in Fig. 1, may be histone substrates or non-histone substrates, consisting of diverse cell cycle-associated enzymes, metabolic enzymes, transcription factors, cell signaling-linked substrates, and structural proteins^9–14^. SIRT2 is expressed in various organs, including the brain, ovary, esophagus, heart, liver, lung, testicles, thyroid, and spleen. Numerous studies have revealed that SIRT2 has a dual function in the formation of malignancies, serving as a tumor promoter or suppressor^15^. Here we focus on SIRT2 as an oncogene. For instance, SIRT2 can encourage the progression of liver cancer through the activation of Akt and subsequent inhibition of epithelial-mesenchymal transition GSK-3, which leads to the raising of the *β*-catenin protein. Additionally, SIRT2 is involved in the activation of genes associated with epithelial-mesenchymal transition (EMT), which reduces intercellular adhesion, fostering aberrant cancer cell proliferation and migration^14^. Additionally, SIRT2 increases the activity of Phosphoenolpyruvate Carboxykinase 1 (PEPCK1) and Glutaminase (GLS), which promote the metabolism of glycolysis and inhibit the E-cadherin pathway, which promotes the invasion of liver cancer cells^16^. Table 1 shows the oncogenic roles of SIRT2 in brain^17,18^, lung^19,20^, gastric^21^, and colon^22^ cancer. The wide range of SIRT2 substrates implies that they are involved in various biological processes^23,24^. Consequently, abnormal SIRT2 activity has been linked to the development and spread of carcinoma maladies^25,26^. That is why SIRT2 is an emerging drug target for therapeutic intervention^14,27,28^. Like all other sirtuins, SIRT2 has two domain structures, the Rossmann fold domain (RFD) and zinc-binding domain (ZBD)^29,30^. SIRT2 binding pocket is situated between the two domains in a wide hydrophobic groove^31,32^. An acetyl-lysine channel and several hydrophobic pockets A-C and a ligand-induced selectivity pocket are present in the SIRT2 active site.

**Table 1:**
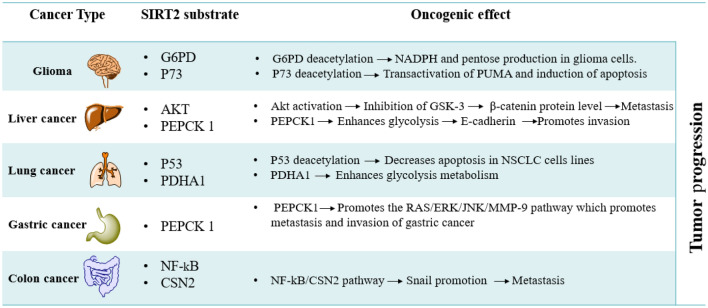
Oncogenic roles of SIRT2 in the progression of various malignancies.

SirReal2 is one of a few selective SIRT2 inhibitors housed in the hydrophobic pocket near the ZBD^32^. The naphthyl group extends into the acetyl-lysine channel, generating van der Waals interactions with nicotinamide and many important residues such as Phe131, Ile169, Leu134, Val233, Ile232, and Phe234 amino acids, where the dimethyl mercaptopyrimidine moiety creates a binding and selectivity pocket^33^.

Computational drug discovery approaches have attracted much attention due to their prospective to speed up the discovery process in respect of time, manpower, and expenses^34^. Computational approaches have been used to effectively design a multitude of novel medications^35^. Historically, most novel therapies have been obtained from natural products (secondary metabolites)^36^. Plant-based medicines make up around a quarter of all FDA-approved medications, including well-known drugs like paclitaxel and morphine^37,38^. In fact, the discovery of drugs from natural products has revolutionized medicine.

**Figure 2:**
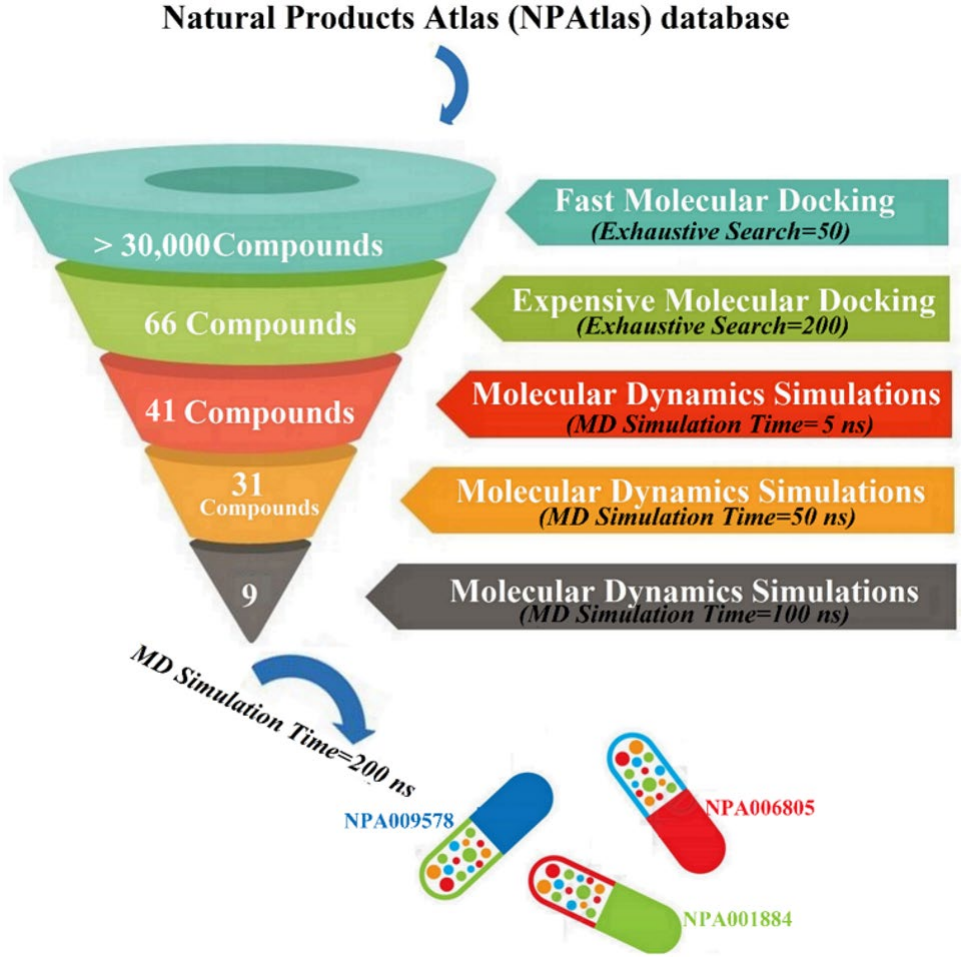
The workflow of the employed in silico approaches for filtration of Natural Products Atlas (NPAtlas) database and identification of potent SIRT2 inhibitors.

In this work, comprehensive in silico approaches were employed to mine the Natural Products Atlas (NPAtlas) database to discover potential SIRT2 inhibitors from a natural source. In accordance with the computed docking scores, the most promising NPAtlas compounds were submitted to molecular dynamics (MD) simulations. The corresponding binding affinities were estimated using the MM-GBSA approach. The constancy of the recognized NPAtlas compounds complexed with SIRT2 was inspected utilizing the structural and energetical investigations during the 200 ns MD course. The workflow of the utilized in silico techniques for filtration of the NPAtlas database and identification of potent SIRT2 inhibitors is illustrated in Fig. 2.

## Results and discussion

### Assessment of docking protocol

**Figure 3:**
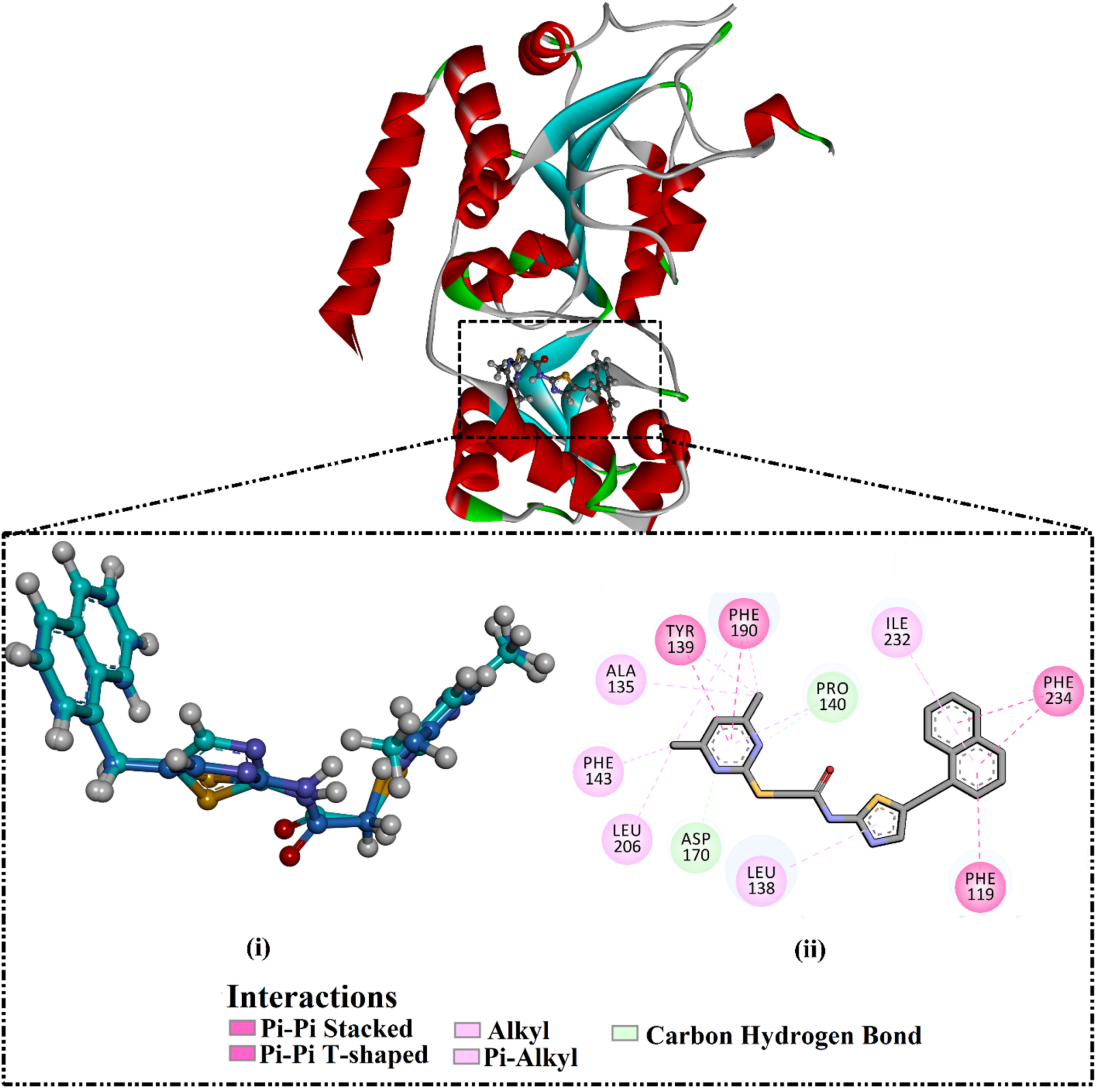
(i) 3D representation of the native structure (in blue) and the anticipated binding mode (in cyan) of SirReal2 and (ii) 2D molecular interaction of the predicted docking pose of SirReal2 inhibitor with SIRT2 protein.

Assessment of the performance of the AutoDock Vina software to portend the correct binding pose of the SIRT2 inhibitors was first evaluated. For assessment, the co-crystallized ligand (i.e., SirReal2) was re-docked towards the SIRT2 protein, and the anticipated docking pose was compared to the experimental binding mode (PDB ID: 4RMG^32^). The anticipated docking pose was almost identical to the native binding mode with an RMSD value of 0.28 Å and a docking score of − 12.0 kcal/mol using expensive docking parameters (Fig. 3). Although the SirReal2 inhibitor was unable to exhibit any hydrogen bond within the binding pocket of the SIRT2 protein, other noncovalent interactions were observed, including π-π stacking interactions with Phe234, Phe119, Tyr139, and Phe190 amino acids (Fig. 3).

In summary, assessment calculations demonstrated the excellent performance of AutoDock Vina software in predicting the binding mode of SIRT2 inhibitors.

### NPAtlas database mining

To explore SIRT2 inhibitors from natural product sources, AutoDock Vina software was utilized to mine the NPAtlas database. Initially, the NPAtlas database was virtually screened against SIRT2 with fast docking parameters. According to the anticipated docking scores, sixty-six NPAtlas compounds unveiled docking scores less than the native inhibitor (SirReal2 = − 11.8 kcal/mol). Thus, those sixty-six NPAtlas compounds were subjected to more reliable docking computations with expensive parameters. The computed docking scores for the identified potent sixty-six NPAtlas compounds are summarized in Table S1. As can be seen from Table S1, only forty-one NPAtlas compounds exhibited lower docking scores than the co-crystalized inhibitor (SirReal2 = − 12.0 kcal/mol). 2D visualization of molecular interactions of those forty-one NPAtlas compounds with the substantial amino acids inside the binding pocket of SIRT2 is shown in Fig. S1. Most of the identified NPAtlas compounds exposed similar SIRT2 docking poses inside the binding pocket of SIRT2, forming fundamental π-π stacking interactions with Phe234, Phe119, Tyr139, and Phe190 amino acids (Fig. S1). 2D chemical structures and computed docking scores for nine potent NPAtlas compounds are given in Table 2. Notably, the listed nine potent NPAtlas compounds in Table 2 were selected based on the estimated binding energies over the 50 ns MD course characterized in the latter sections.

**Table 2:** 2D chemical structures and evaluated docking scores (in kcal/mol) for SirReal2 and nine promising NPAtlas compounds towards SIRT2 protein.

No	NPAtlas code	2D-Chemical structure	Docking score (kcal/mol)^a^	
			Fast	Expensive
	SirReal2		− 11.8	− 12.0
1	NPA009578		− 13.3	− 13.4
2	NPA006805		− 13.1	− 13.2
3	NPA001884		− 13.0	− 13.1
4	NPA023712		− 13.0	− 12.9
5	NPA000470		− 12.9	− 12.9
6	NPA018719		− 12.7	− 12.8
7	NPA023225		− 12.6	− 12.5
8	NPA014478		− 12.5	− 12.4
9	NPA001597		− 12.6	− 12.2

^a^Data ranked in accordance with the expensive docking scores.

**Figure 4:**
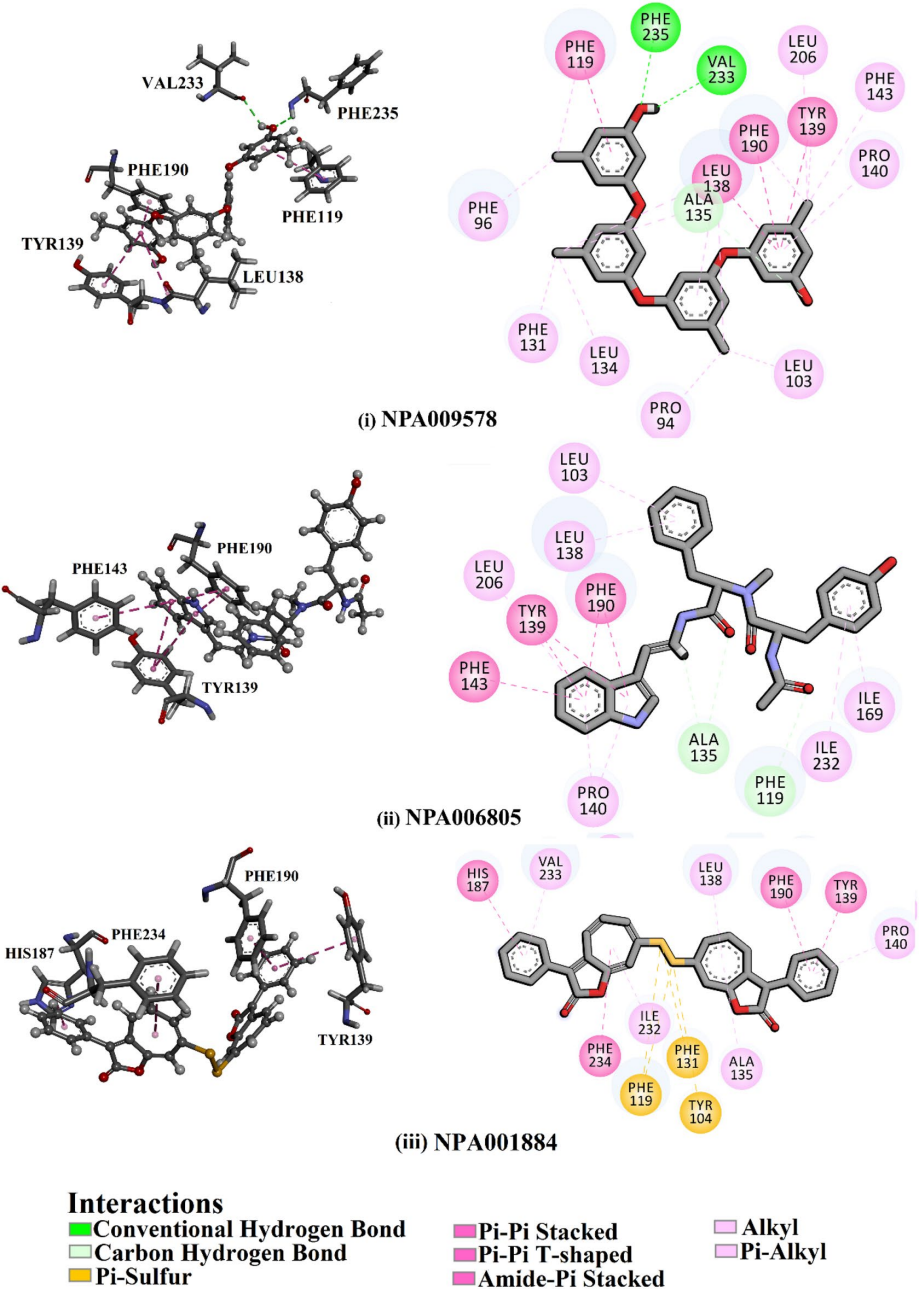
Three- and two-dimensional representations of the anticipated docking poses of (i) NPA009578, (ii) NPA006805, and (iii) NPA001884 against SIRT2 protein, utilizing expensive docking parameters.

According to data listed in Table 2, NPA009578, NPA006805, and NPA001884 manifested solid binding affinities toward SIRT2 protein with docking scores of about − 13.2 kcal/mol. NPA009578 (Tetraorcinol A), separated from an *Aspergillus versicolor*, unveiled the greatest binding affinity toward SIRT2 protein with a docking score of − 13.4 kcal/mol (Table 2). Investigating the predicted docking pose of NPA009578 revealed that the OH of the m-cresol ring participated in two hydrogen bonds with CO of Val233 and NH of Phe235 with bond lengths of 2.48 and 2.63 Å, respectively (Fig. 4). Furthermore, the two benzene rings of two m-cresol rings exhibited two π-π stacking, amide-π stacking, and π-π T-shaped interactions with Phe119, Leu138, Tyr39, and Phe190 (Fig. 4).

NPA006805 (Miyakamide B1), segregated from *Aspergillus flavus*, exposed the second eminent docking score against SIRT2 protein with a value of − 13.2 kcal/mol (Table 2). The indole ring of NPA006805 demonstrated four π-π stacking and one π-π T-shaped interaction with Phe190, Tyr139, and Phe143 (Fig. 4).

NPA001884 (Roseobacticide J), isolated from *Phaeobacter gallaeciensis*, also manifested good binding affinity against SIRT2 protein with a docking score of − 13.1 kcal/mol (Table 2). For NPA001884, the two benzene rings and cyclohepta-1,3,5-triene formed two π-π stacking and two π-π T-shaped interactions with Tyr139, His187, Phe190, and Phe234 (Fig. 4).

According to the unveiled binding modes of the identified NPA009578, NPA006805, and NPA001884, these natural compounds share a similar pharmacophore that includes two aromatic rings diverged by approximately 10 Å away from each other^8^ (Fig. S2).

### Molecular dynamics simulations

**Figure 5:**
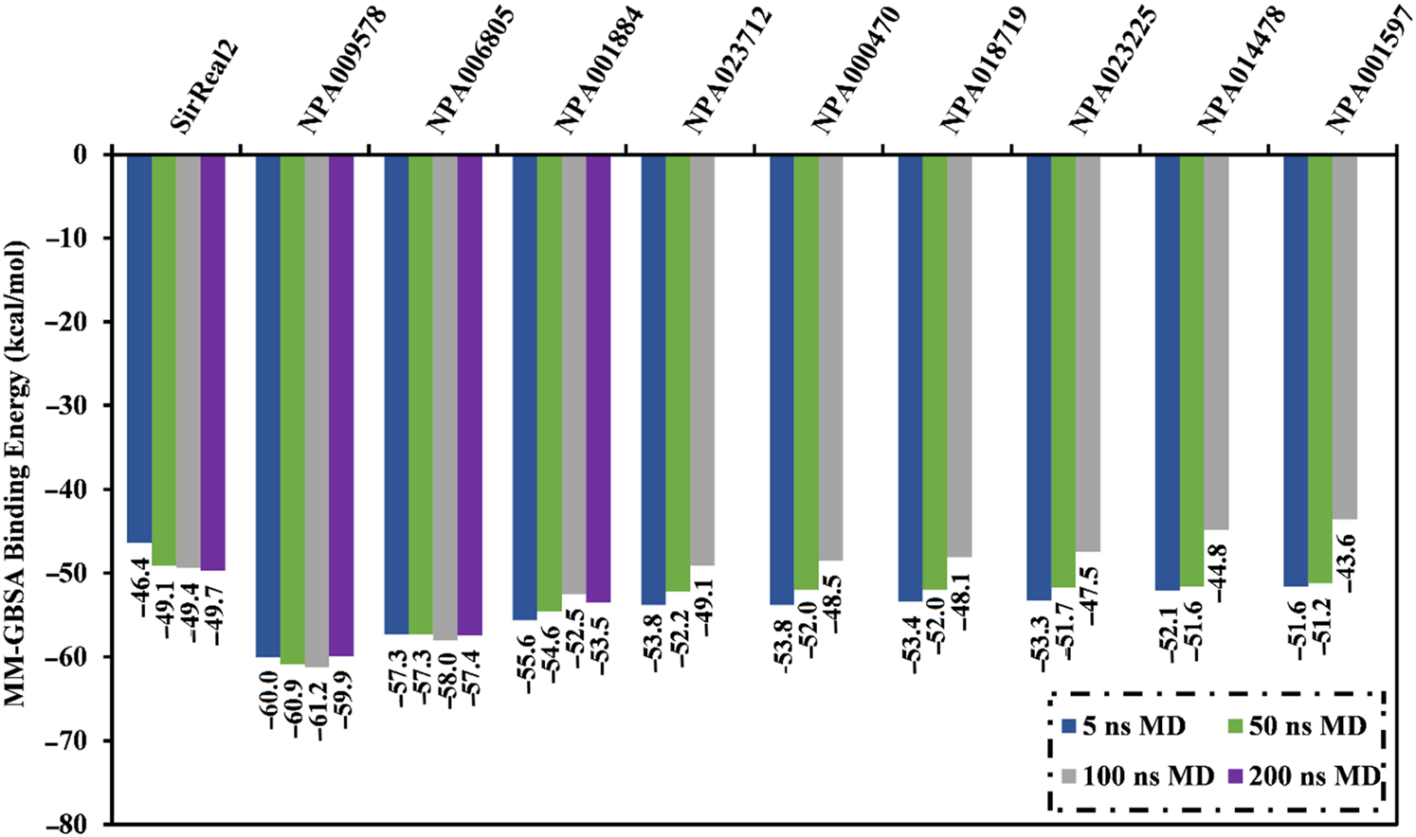
Average binding energies for the SirReal2 inhibitor and the identified potent NPAtlas compounds in complex with SIRT2 protein over 5 ns, 50 ns, 100 ns, and 200 ns MD simulations.

MD simulations probe the steadiness of the target-ligand complexes, the trustworthiness of target-ligand affinities, structural specifics, and conformational elasticities^39,40^. Consequently, the most potent NPAtlas molecules with docking scores less than the native ligand (SirReal2 = − 12.0 kcal/mol) were submitted to MD simulations, pursued by binding energy computations. To lessen time and in silico costs, the MDs were executed for a short simulation time of 5 ns. The corresponding binding affinities were evaluated and listed in Table S1. As listed in Table S1, thirty-one NPAtlas molecules revealed lower binding energies (Δ*G*_binding_) compared to the native ligand SirReal2 (calc. − 46.4 kcal/mol). Consequently, those thirty-one NPAtlas compounds were nominated and submitted to MD simulation throughout 50 ns to acquire more accurate binding affinities. The binding affinities were computed and summarized in Table S2. As demonstrated in Table S2, nine NPAtlas molecules unveiled lower binding energies (Δ*G*_binding_) compared to the co-crystallized SirReal2 inhibitor (calc. − 49.1 kcal/mol). Therefore, those nine potent NPAtlas molecules were opted and subjected to 100 ns MD simulations. As well, the corresponding binding affinities were computed and depicted in Fig. 5. Figure 5 reveals that three out of those nine NPAtlas compounds, namely NPA009578, NPA006805, and NPA001884, demonstrated less binding energies (Δ*G*_binding_) compared to the co-crystallized SirReal2 ligand (calc. − 49.4 kcal/mol). The computed MM-GBSA binding energies for NPA009578, NPA006805, and NPA001884 against SIRT2 were − 61.2, − 58.0, and − 52.5 kcal/mol throughout the MD simulation time of 100 ns, respectively (Fig. 5). MD simulations were prolonged to 200 ns for those three potent NPAtlas compounds complexed with SIRT2 protein to achieve more dependable MM-GBSA binding energies (Fig. 5).

Notably, there is no remarkable unevenness between the estimated binding affinities over 100 and 200 ns MD simulations for NPA009578, NPA006805, and NPA001884 complexed with SIRT2 protein (Fig. 5). Compared to the binding energy of SirReal2 (calc. − 49.7 kcal/mol), NPA009578, NPA006805, and NPA001884 displayed lower binding energy towards SIRT2 over the simulation time of 200 ns with average Δ*G*_binding_ values of − 59.9, − 57.4, and − 53.5 kcal/mol, respectively (Fig. 5).

To recognize the driving force in the binding of NPA009578, NPA006805, NPA001884, and SirReal2 with SIRT2, the binding affinities were broken down into their physical parts (Table 3). Based on the energy decomposition data, *E*_vdw_ was noticed to be the considerable participator prompting molecular complexation with SIRT2 for NPA009578 (Δ*E*_vdw_ of − 76.2 kcal/mol), NPA006805 (Δ*E*_vdw_ of − 67.0 kcal/mol), NPA001884 (Δ*E*_vdw_ of − 73.7 kcal/mol), and SirReal2 (Δ*E*_vdw_ of − 64.0 kcal/mol). *E*_ele_ was ditto favorable with an average value of − 7.4, − 13.5, − 33.5, and − 6.6 kcal/mol for NPA009578-, NPA006805-, NPA001884-, and SirReal2-SIRT2 binding energies, respectively (Table 3).

**Table 3:** Physical parts of the estimated binding energies for NPA009578-, NPA006805-, NPA001884-, and SirReal2-SIRT2 complexes as specified via MD simulation over 200 ns.

NPAtlas code	Evaluated MM-GBSA binding energy (kcal/mol)						
	∆*E*_vdw_	∆*E*_GB_	∆*E*_ele_	∆*G*_gas_	∆*E*_SUR_	∆*G*_Solv_	∆*G*_binding_
SirReal2	− 64.6	28.9	− 6.6	− 71.2	− 7.4	21.5	− 49.7
NPA009578	− 76.2	32.2	− 7.4	− 83.6	− 8.6	23.7	− 59.9
NPA006805	− 67.0	31.0	− 13.5	− 80.5	− 7.9	23.1	− 57.4
NPA001884	− 73.7	62.1	− 33.5	− 107.3	− 8.3	53.7	− 53.5

**Figure 6:**
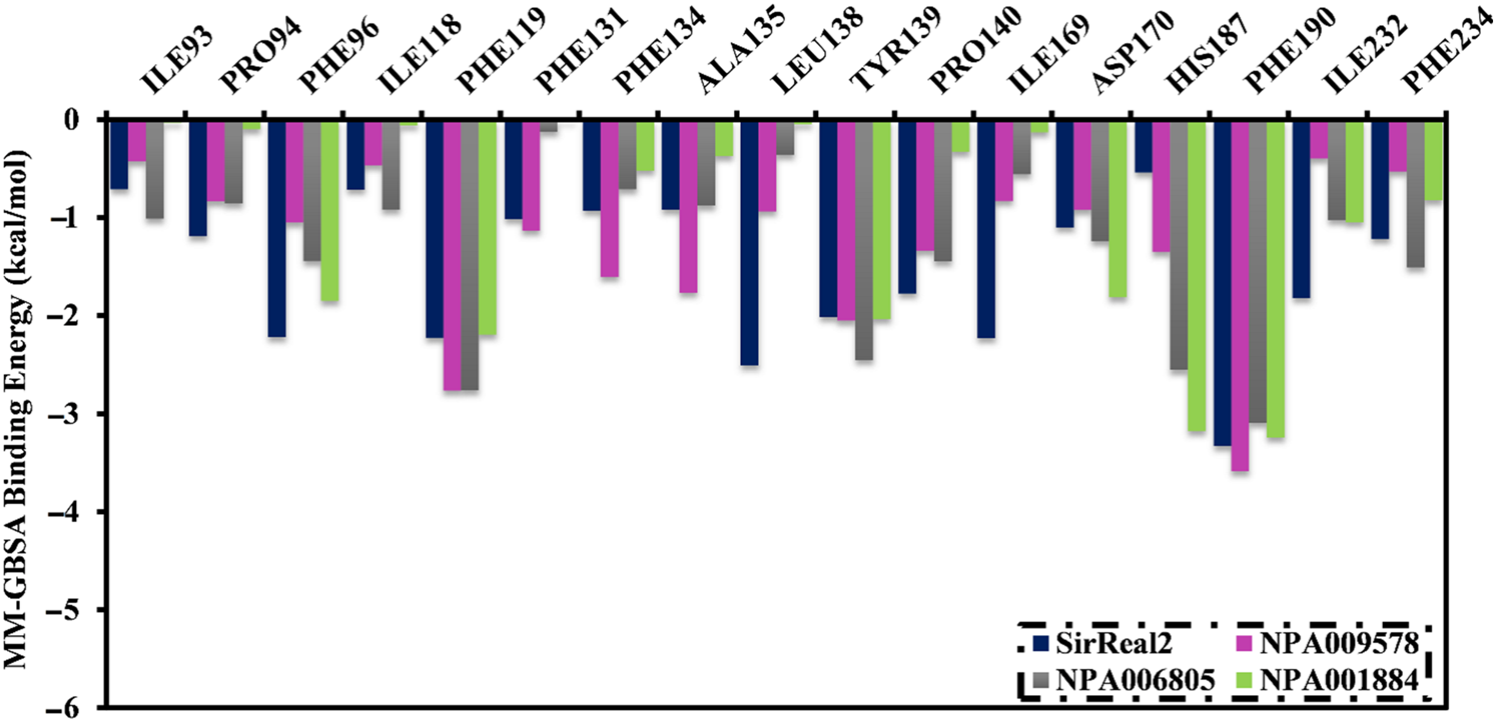
Energy contributions of the essential amino acids to the total MM-GBSA binding energy (kcal/mol) of SirReal2, NPA009578, NPA006805, and NPA001884 in complex with SIRT2 protein.

The binding affinities of the inspected NPAtlas molecules complexed with SIRT2 protein were then decomposed at the per-residue level, and the amino acids with energy participation less than − 0.50 kcal/mol were demonstrated (Fig. 6). As shown in Fig. 6, Phe119, Tyr139, Phe190, and Phe234 favorably contribute to the binding of NPA009578, NPA006805, NPA001884, and SirReal2 with SIRT2 protein. Phe190 shared the overall MM-GBSA binding energies outstandingly with values of − 3.5, − 3.1, − 3.2, and − 3.3 kcal/mol for NPA009578-, NPA006805-, NPA001884-, and SirReal2-SIRT2 complexes, respectively (Fig. 5). Phe119 was the second-greatest participator in the total MM-GBSA binding energies with values of − 2.7, − 2.6, − 2.2, and − 2.2 kcal/mol for NPA009578-, NPA006805-, NPA001884-, and SirReal2-SIRT2 complexes, respectively (Fig. 6). Notably, all inspected systems have almost identical interaction modalities with proximal residues, which points out a similarity in the binding pose of the identified inhibitors inside the binding pocket of the SIRT2 protein.

### Post-dynamics analyses

To further inspect the steadiness of NPA009578, NPA006805, and NPA001884 in complex with SIRT2 protein, energetical and structural investigations were conducted over the 200 ns MD course and emulated to those of the native SirReal2 ligand. Observing the structural and energetical stabilization of the inspected complexes was realized by examining binding energy per trajectory, center-of-mass (CoM) distance, root-mean-square deviation (RMSD), root-mean-square fluctuation (RMSF), and radius of gyration (Rg).

#### Binding energy per trajectory

**Figure 7:**
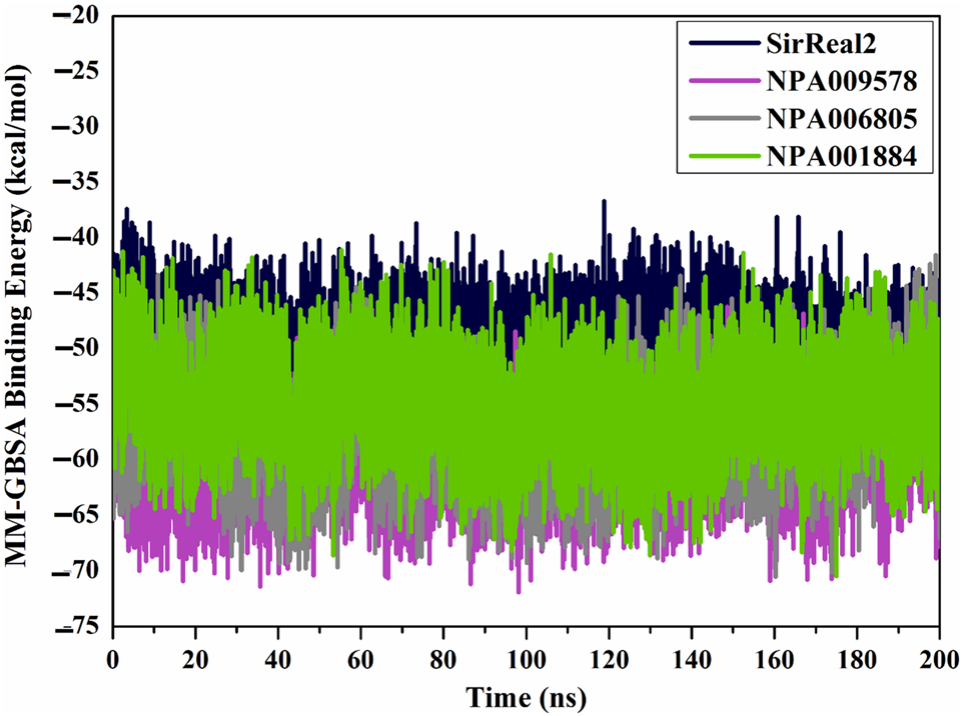
Computed binding energy per trajectory for SirReal2 (in navy), NPA009578 (in pink), NPA006805 (in gray), and NPA001884 (in green) complexed with SIRT2 protein throughout 200 ns MD simulations.

The structural constancy of NPA009578, NPA006805, NPA001884, and SirReal2 complexed with SIRT2 was inclusively evaluated throughout a 200 ns MD simulation by gauging the correlation between binding energy and time (Fig. 7). The most intriguing portion of this graph is the entire immutability for NPA009578, NPA006805, NPA001884, and SirReal2 complexed with SIRT2 protein with average Δ*G*_binding_ of − 59.9, − 57.4, − 53.5, and − 49.7 kcal/mol, respectively. On the basis of this binding energy per trajectory, all scrutinized complexes preserved stabilization over the 200 ns MD course.

#### Root-mean-square deviation

**Figure 8:**
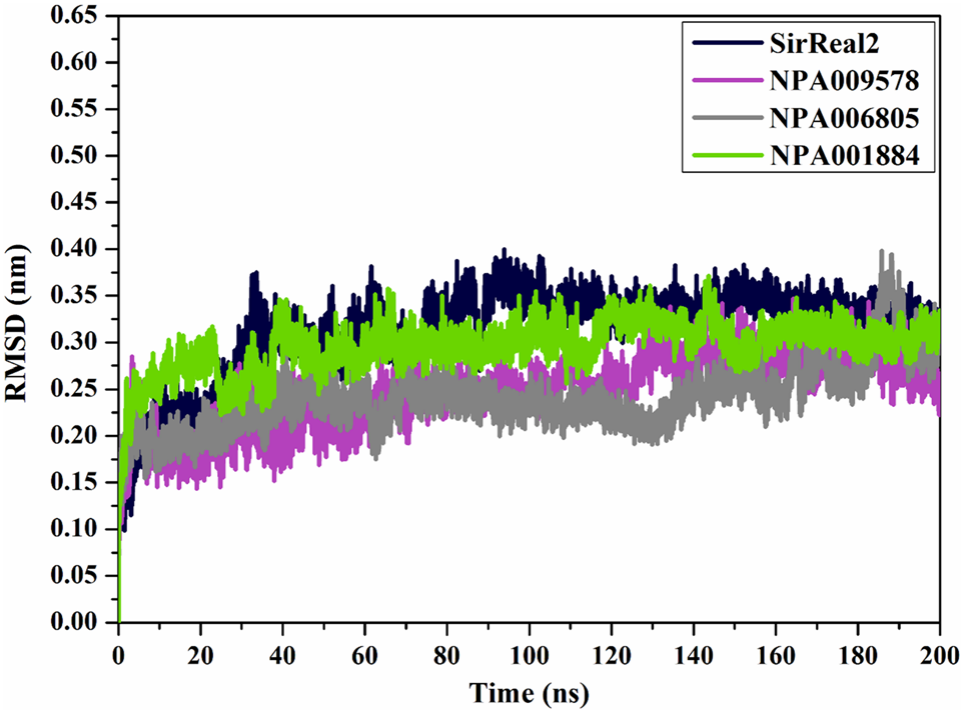
RMSD plot for the backbone with respect to the starting coordinates of SirReal2 (in navy), NPA009578 (in pink), NPA006805 (in gray), and NPA001884 (in green) complexed with SIRT2 protein over 200 ns MD simulations.

To interpret the positional and conformational changes in the identified NPAtlas compounds in complex with SIRT2 protein, the root-mean-square deviation (RMSD) of the backbone atoms from the simulation trajectories was measured (Fig. 8). Interestingly, the measured RMSD values for the inspected NPAtlas compounds with SIRT2 protein were maintained beneath 0.3 nm over 200 ns MD simulations (Fig. 8). These findings emphasize that the identified NPAtlas compounds are strongly bound in the binding pocket and have no impact on the overall structure of the SIRT2 protein.

#### Center-of-mass distance

**Figure 9:**
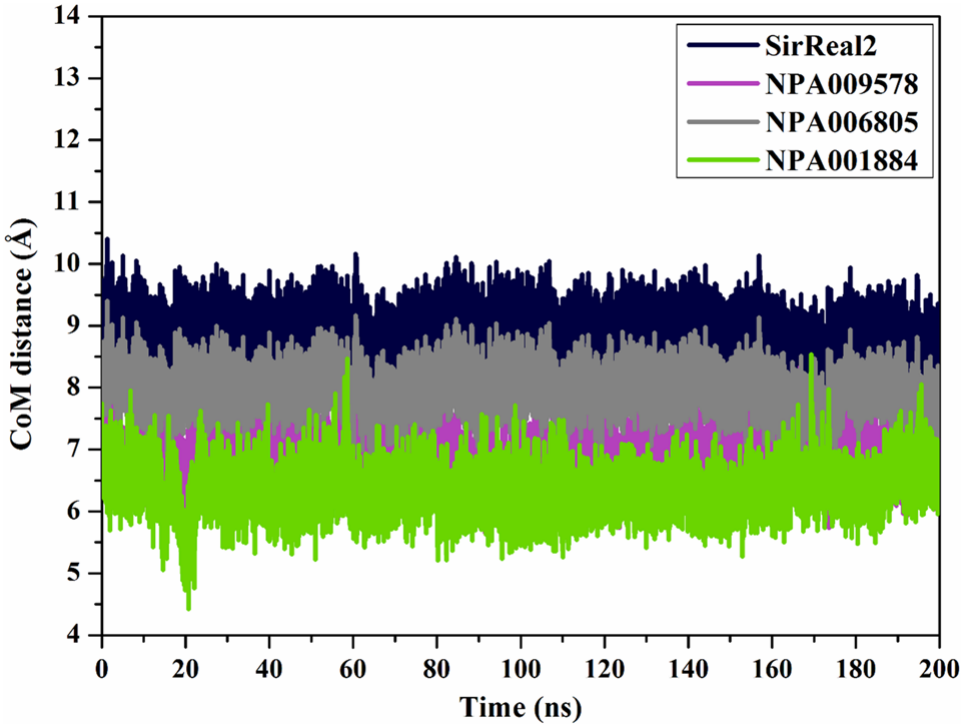
CoM distances (in Å) between SirReal2 (in navy), NPA009578 (in pink), NPA006805 (in gray), and NPA001884 (in green) and Phe190 of the SIRT2 throughout 200 ns MD simulation.

The center-of-mass (CoM) distance was measured between the NPAtlas compounds and Phe190 residue to provide a deeper insight into the constancy of NPAtlas-SIRT2 complexes over 200 ns MD course (Fig. 9). The graph demonstrates that the measured CoM distances were steady for NPA009578-, NPA006805-, NPA001884-, and SirReal2-SIRT2 complexes with average values of 6.9, 7.9, 6.3, and 8.7 Å, respectively (Fig. 9). This finding established the perfect steady of the identified NPAtlas compounds complexed with SIRT2 over 200 ns MD course.

#### Root-mean-square fluctuation

**Figure 10:**
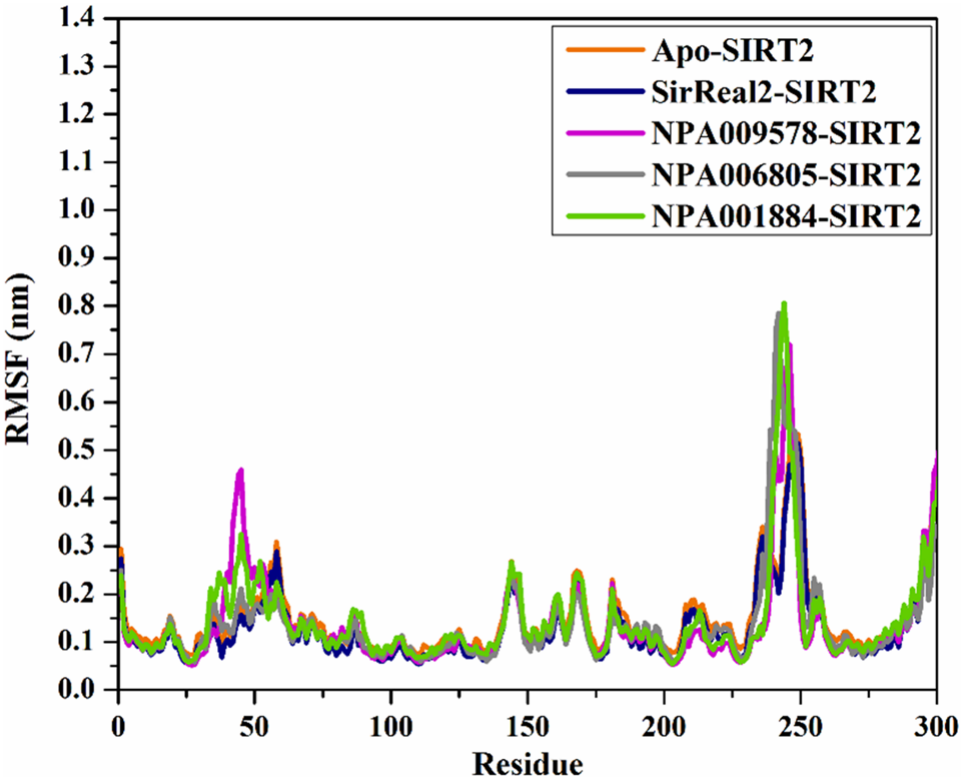
RMSF of the C_α_ atoms of apo and ligand-soaked SIRT2 protein throughout 200 ns MD simulation. Color Scheme: Apo SIRT2 (in orange), SirReal2-SIRT2 (in navy), NPA009578-SIRT2 (in pink), NPA006805-SIRT2 (in gray), and NPA001884-SIRT2 (in green).

To determine the backbone conformational variation and stability of the backbone of the apo SIRT2, NPA009578-SIRT2, NPA006805-SIRT2, NPA001884-SIRT2, and SirReal2-SIRT2 complexes, the root-mean-square fluctuation (RMSF) of C_α_ was inspected and illustrated in Fig. 10. As depicted in Fig. 10, the residues were found stationary in the NPA009578-SIRT2, NPA006805-SIRT2, NPA001884-SIRT2, and SirReal2-SIRT2 complexes over the 200 ns MD simulations.

#### Radius of gyration

**Figure 11:**
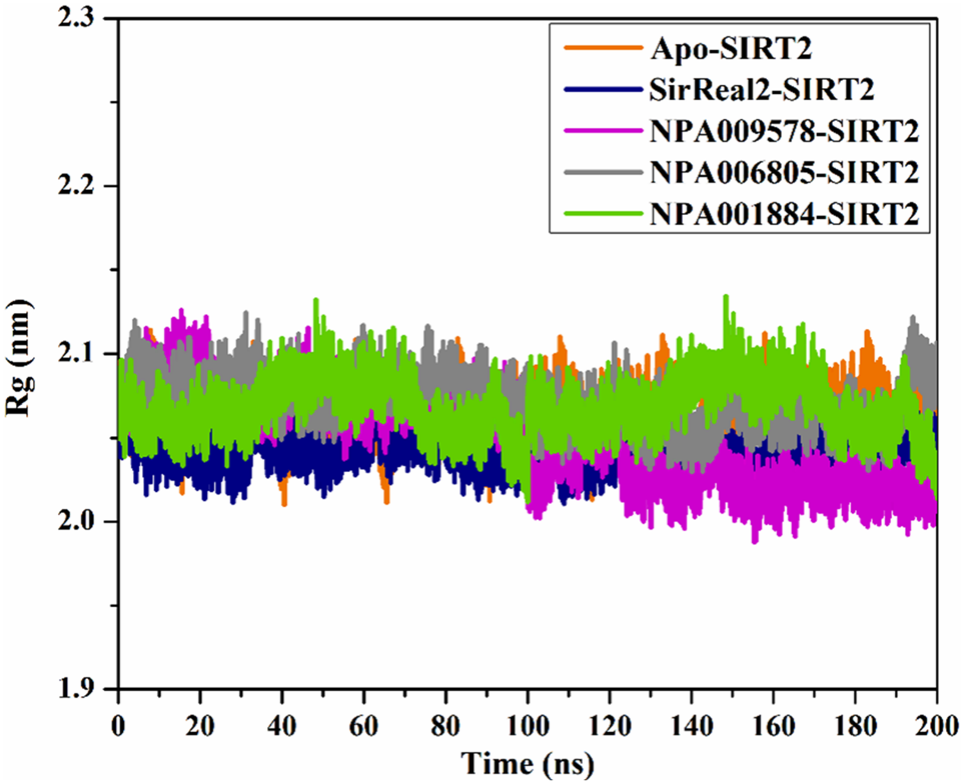
Radius of gyration (Rg) of apo and ligand-soaked SIRT2 protein throughout 200 ns MD simulation. Color Scheme: Apo SIRT2 (in orange), SirReal2-SIRT2 (in navy), NPA009578-SIRT2 (in pink), NPA006805-SIRT2 (in gray), and NPA001884-SIRT2 (in green).

The radius of gyration (Rg) was pinpointed to notice the compactness of SIRT2 in the apo and complexed with identified NPAtlas compounds over the 200 ns MD simulation. Rg analysis provided the comprehensive folding and unfolding of SIRT2 structure upon binding with NPAtlas compounds. The average Rg values were 2.06¸ 2.04, 2.04, 2.07, and 2.05 nm for apo-SIRT2, SirReal2-SIRT2, NPA009578-SIRT2, NPA006805-SIRT2, and NPA001884-SIRT2, respectively, as depicted in Fig. 11. The Rg analysis displayed that SIRT2 remains compact upon binding with SirReal2, NPA009578, NPA006805, and NPA001884 over the 200 ns MD simulations. These findings proved that binding of SirReal2, NPA009578, NPA006805, and NPA001884 considerably stabilized the SIRT2 structure.

### Drug-likeness features

The effectuality of medicinal remedies is appreciably dependent on the molecular properties and bioactivity of drug candidates^41^. To anticipate the drug-like features and bioactivity of the inspected NPAtlas compounds as SIRT2 inhibitors, a SwissADME webserver was utilized. The estimated physiochemical characteristics are depicted in Table 4. As can be seen from the data listed in Table 4, promising physiochemical characteristics were noticed, except NPA001884. The later NPAtlas compound demonstrated infringements in a few features like molecular weight (MW) and Mlog*P*. The Mlog*P* of NPA009578, NPA006805, and SirReal2 were auspicious, with values ≤ 5^42^. The TPSA values of NPA009578, NPA006805, NPA001884, and SirReal2 were < 140 Å^2^, disclosing that those identified inhibitors have excellent membrane permeability or oral absorption^43^. Besides, the number of hydrogen bond donors (HBD) was ≤ 5. The number of hydrogen bond acceptors (HBA) was ≤ 10. The molecular weights (MW) for NPA009578, NPA006805, NPA001884, and SirReal2 were 442.50, 524.61, 506.59, and 420.55 g/mol, respectively (Table 4). Notably, this little elevation in MW will not have a striking influence on inhibitor diffusion and transmission, wherever it has been reported that several FDA-approved drugs have MW greater than 500 g/mol^44^.

**Table 4:** Anticipated physiochemical characteristics of SirReal2 and the inspected NPAtlas compounds as promising SIRT2 inhibitors.

NPAtlas Code	Mlog*P*	HBA	Nrotb	HBD	TPSA	MW	%ABS
SirReal2	2.65	4	7	1	121.31	420.55	67.1%
NPA009578	4.67	5	6	2	68.15	442.50	85.5%
NPA006805	1.86	4	13	4	114.53	524.61	69.5%
NPA001884	5.11	4	5	0	111.02	506.59	70.7%

## Conclusion

In a slew of studies, sirtuin 2 (SIRT2) has been linked to cancer pathogenesis. As a result, SIRT2 inhibition stands out as a possible intervention strategy in the long-running fight against cancer. Hence, in silico-based techniques were performed to identify potential natural products from the NPAtlas database that might inhibit the activity of the SIRT2 protein. Based on the molecular docking, molecular dynamics, and MM-GBSA binding energy results, NPA009578, NPA006805, and NPA001884 were identified as prospective SIRT2 inhibitors. According to the estimated binding affinities over 200 ns MD simulations, NPA009578, NPA006805, and NPA001884 demonstrated better binding affinities compared to the co-crystallized SirReal2 with Δ*G*_binding_ values of − 59.9, − 57.4, − 53.5, and − 49.7 kcal/mol, respectively. The energetical and structural inspections throughout 200 ns MD simulations indicated great stabilization for the identified NPAtlas compounds complexed with SIRT2 protein. Besides, the identified NPAtlas compounds manifested convenient drug-like features and oral bioavailability. These observations imply that NPA009578, NPA006805, and NPA001884 might be promising SIRT2 inhibitors that deserve further in vitro and in vivo examinations.

## Computational methodology

### SIRT2 preparation

All in silico computations utilized the X-ray resolved three-dimensional (3D) structure of the human SIRT2 protein complexed with SirReal2 (PDB code: 4RMG, resolution: 1.88 Å)^32^. The SIRT2 structure was prepared by removing all heteroatoms, including crystallographic waters, ions, and ligand. H++ server was used to deduce the protonation states of the titratable amino acids.

### Database preparation

The Natural Products Atlas (NPAtlas) database was downloaded in SDF format and prepared for in silico drug discovery calculations^45^. Using the Omega2 software^46,47^, the 3D chemical structures of NPAtlas compounds were created. The generated 3D chemical structures were optimized by the MMFF94S force field inside the SZYBKI program^48,49^. The protonation state of the molecules was investigated utilizing fixpka application, implemented inside the QUACPAC software^50^. Duplicated NPAtlas compounds with identical international chemical identifier keys (InChIKey) were eliminated^51^. The prepared NPAtlas compounds in mol2 format can be downloaded from www.compchem.net/ccdb.

### Molecular docking

Two stages of molecular docking computations, namely fast and expensive, were conducted with the assistance of AutoDock Vina software^52^. According to AutoDock protocol^53^, the SIRT2 structure was saved in pdbqt format utilizing the MGTools program. In the current study, two levels of docking accuracy were employed, namely fast and expensive docking calculations. The exhaustiveness number was 50 and 200 for fast and expensive docking computations, respectively. At the same time, the remaining settings were kept to their default values. A grid box with dimensions of 20 Å × 20 Å × 20 Å, with a grid spacing value of 1.0 Å, was utilized to embrace the binding pocket of SIRT2 protein. The grid center was located at the following coordinates: *x* = 130.869, *y* = 126.675, and *z* = 145.206.

### Molecular dynamics simulations

AMBER16 software was applied to run molecular dynamics (MD) simulations for the most promising NPAtlas compounds complexed with SIRT2 protein^54^. AMBER force field 14SB was utilized to characterize the SIRT2 protein^55^. On the other hand, the General AMBER force field (GAFF2) was used to describe the identified NPAtlas molecules^56^. Using Gaussian09 software^57^, the charges were estimated at the HF/6-31G* level using the restrained electrostatic potential (RESP) fitting approach^58^. TIP3P water molecules in a truncated octahedral box were added to solvate the NPAtlas-SIRT2 complexes, with a spacing of 12 Å between the solute and the box edge^59^. Sufficient number of sodium and chloride ions were employed to neutralize the solvated complex. A salt concentration of 0.15 M NaCl was maintained. The solvated NPAtlas-SIRT2 complexes were subjected to 5000 steps of energy minimization. After that, the minimized systems were progressively annealed up to 300 K throughout 50 ps. An equilibration stage of 10 ns was executed under isobaric-isothermal (NPT) ensemble. Ultimately, the production runs were executed throughout 5, 50, 100, and 200 ns MD simulations. Snapshots of the MD trajectories were recorded every 10 ps. Non-bonded interactions were truncated with a cutoff distance of 12 Å. The electrostatic interactions were handled utilizing the particle-mesh Ewald (PME) method^60^. The temperature of NPAtlas-SIRT2 complexes was preserved at 298 K via the Langevin thermostat. The pressure was managed using the Berendsen barostat^61^. The SHAKE algorithm with an integration step of 2 fs was utilized to restrict all bonds containing hydrogen atoms. All MD simulations were carried out using the GPU version of pmemd (pmemd.cuda) within AMBER16 software. The CompChem GPU/CPU hybrid cluster (hpc.compchem.net) was used for all in silico computations. All molecular interactions were visualized by the Discovery Studio module of Biovia software^62^.

### MM-GBSA binding energy

To compute the binding energies of the inspected NPAtlas molecules complexed with SIRT2 protein, the molecular mechanic-generalized Born surface area (MM-GBSA) approach was used^63^. The average binding free energy (Δ*G*_binding_) was calculated on the basis of the equations demonstrated below:

$$\Delta G_{{{\mathrm{binding}}}} = G_{{{\mathrm{NPAtlas}} - {\mathrm{SIRT}}2 }} - \left( {G_{{{\mathrm{NPAtlas}}}} + G_{{{\mathrm{SIRT}}2}} } \right)$$

where the energy term (*G*) is computed as:

$$G = G_{{{\mathrm{GB}}}} + E_{{{\mathrm{vdw}}}} + G_{{{\mathrm{SA}}}} + E_{{{\mathrm{ele}}}}$$

*E*_ele_ symbolizes electrostatic energy. *E*_vdw_ indicates van der Waals energy. *G*_GB_ and *G*_SA_ stand for nonpolar and polar participation of the solvation-free energy, respectively. The Generalized Born (GB) model was utilized to compute *G*_GB_ using the parameters suggested by Onufriev et al. (igb = 2)^64^. *G*_SA_ was evaluated according to the solvent-accessible surface area (SASA) using the LCPO algorithm^65^. The contribution of conformation entropy was neglected because of its great computation cost and low anticipation thoroughness^66,67^.

### Drug-likeness characteristics

Using the SwissADME webserver (http://www.swissadme.ch/), physicochemical characteristics were evaluated for the most potent NPAtlas compounds. The estimated characteristics included HBA (hydrogen bond acceptor), MW (molecular weight), Mlog*P* (octanol/water partition coefficient), HBD (hydrogen bond donor), %ABS (percent absorption), RB (rotatable bond count), and TPSA (topological polar surface area). According to Lipinski’s rule of five (RO5), the orally active compounds should contain no more than 5 HBD and 10 HBA. Besides, Mlog*P*, MW, and TPSA of the orally bioavailable compounds should be less than 5, 500, and 140 Å^2^, respectively. %ABS was computed as follows^68^:

$$\% {\mathrm{ABS}} = {1}0{9}{-}\left[ {0.{345 } \times {\text{ TPSA}}} \right]$$

## Supplementary Information

Supplementary Information.

## Data Availability

The generated and analyzed data during the current study is supplied in this manuscript and supplementary material.
