# Supplementary material for: In silico drug discovery of SIRT2 inhibitors from natural source as anticancer agents
Source: Sci Rep. 2023 Feb 7;13:2146. doi: 10.1038/s41598-023-28226-7 (PMC9905574; doi:10.1038/s41598-023-28226-7)
Supplement: Supplementary file 1 — Supplementary Information. [file 41598_2023_28226_MOESM1_ESM.docx]

***In Silico Drug Discovery of SIRT2 Inhibitors from Natural Source as Anticancer Agents***

Mahmoud A. A. Ibrahim ^1*^, Khlood A. A. Abdeljawaad ^1^, Eslam Roshdy ^2^, Dina E. M. Mohamed ^1^, Taha F. S. Ali ^2,3^, Gamal A. Gabr ^4,5^, Laila A. Jaragh-Alhadad ^6^, Gamal A. H. Mekhemer ^1^, Ahmed M. Shawky ^7^, Peter A. Sidhom ^8^, Alaa H. M. Abdelrahman ^1^

*^1^Computational Chemistry Laboratory, Chemistry Department, Faculty of Science, Minia University, Minia 61519, Egypt; m.ibrahim@compchem.net,* [*kh.abdeljawaad@compchem.net*](mailto:kh.abdeljawaad@compchem.net)*,* [*d.mohamed@compchem.net*](mailto:d.mohamed@compchem.net)*, gmekhemer@mu.edu.eg, a.abdelrahman@compchem.net*

*^2^Medicinal Chemistry Department, Faculty of Pharmacy, Minia University, Minia, 61519, Egypt; m211749@hiroshima-u.ac.jp, taha.ali@mu.edu.eg*

*^3^Department of Chemistry, Graduate School of Science, Hiroshima University, Higashi-Hiroshima, Hiroshima 739-8526, Japan*

*^4^Department of Pharmacology and Toxicology, College of Pharmacy, Prince Sattam Bin Abdulaziz University, Al-Kharj 11942, Saudi Arabia; g.gabr@psau.edu.sa*

*^5^Agricultural Genetic Engineering Research Institute (AGERI), Agricultural Research Center, Giza, Egypt*

*^6^Department of Chemistry, Faculty of Science, Kuwait University, Safat 13060, Kuwait; laila.alhadad@ku.edu.kw*

*^7^Science and Technology Unit (STU), Umm Al-Qura University, Makkah 21955, Saudi Arabia;* [*amesmail@uqu.edu.sa*](mailto:amesmail@uqu.edu.sa)

*^8^Department of Pharmaceutical Chemistry, Faculty of Pharmacy, Tanta University, Tanta 31527, Egypt; peter.ayoub@pharm.tanta.edu.eg*

**
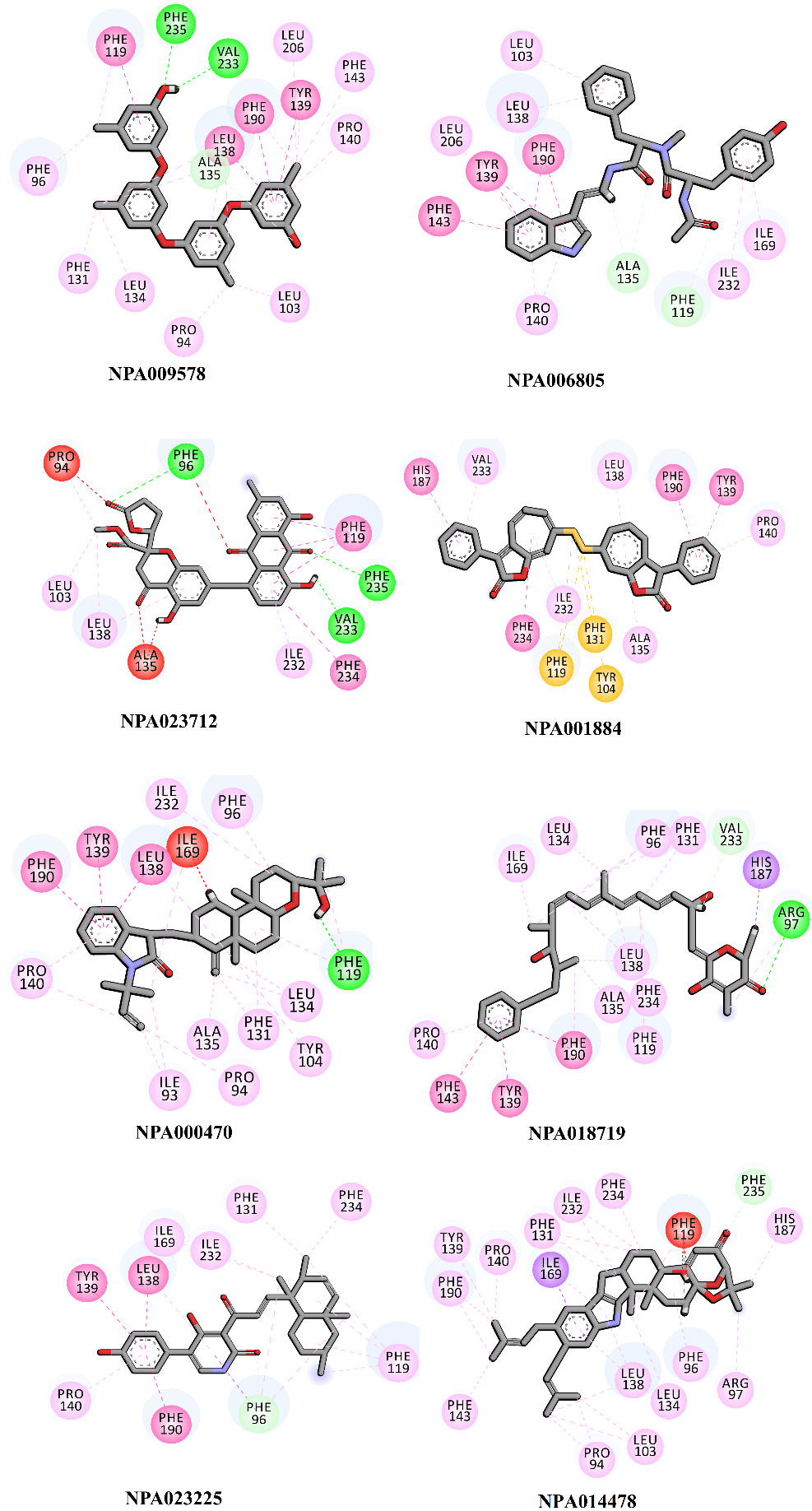
**

**Figure S1.** 2D representations of interactions of the top forty-one potent NPAtlas compounds with important amino acid residues of SIRT2 active site.


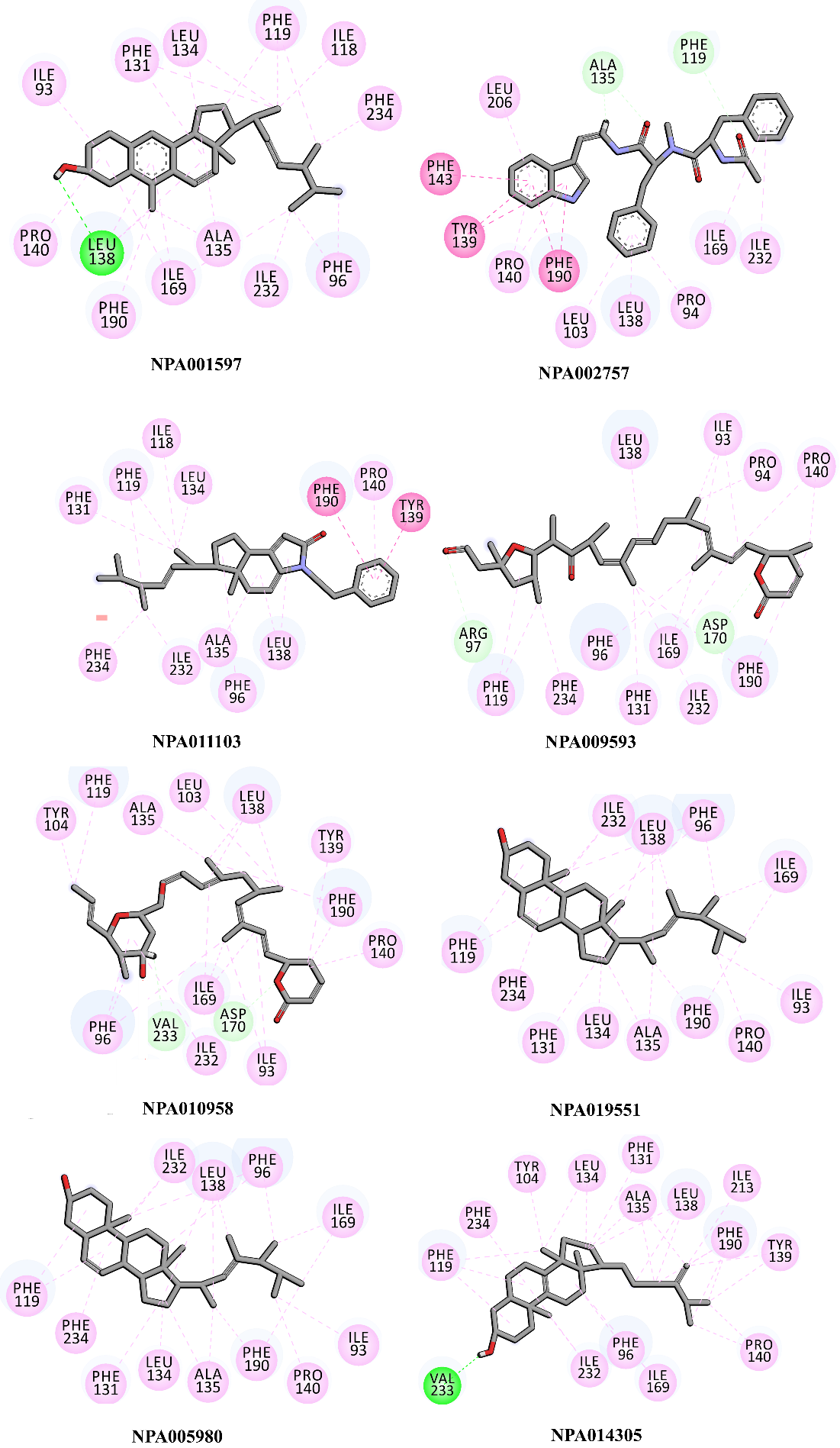


**Figure S1.** *Continued.*


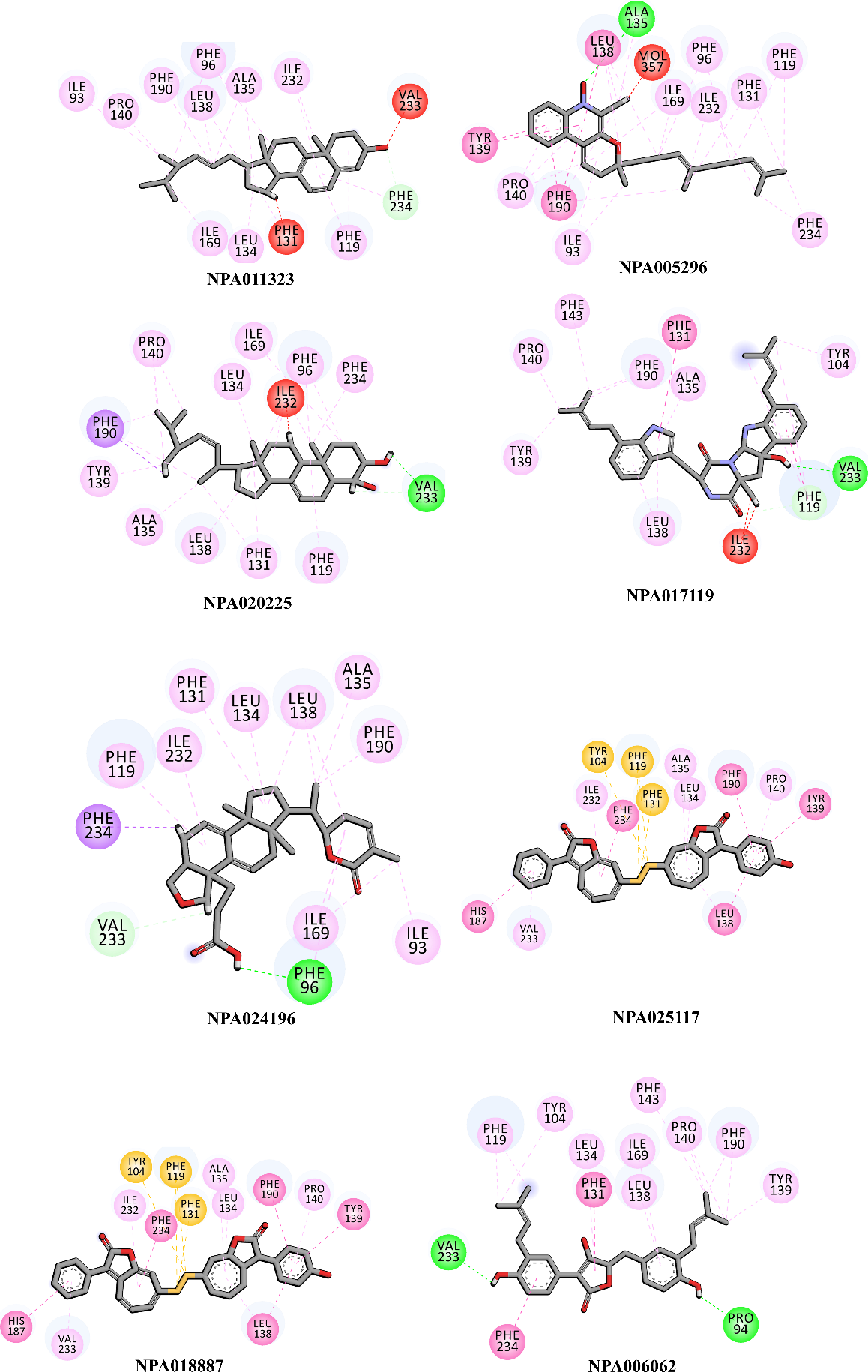


**Figure S1.** *Continued.*


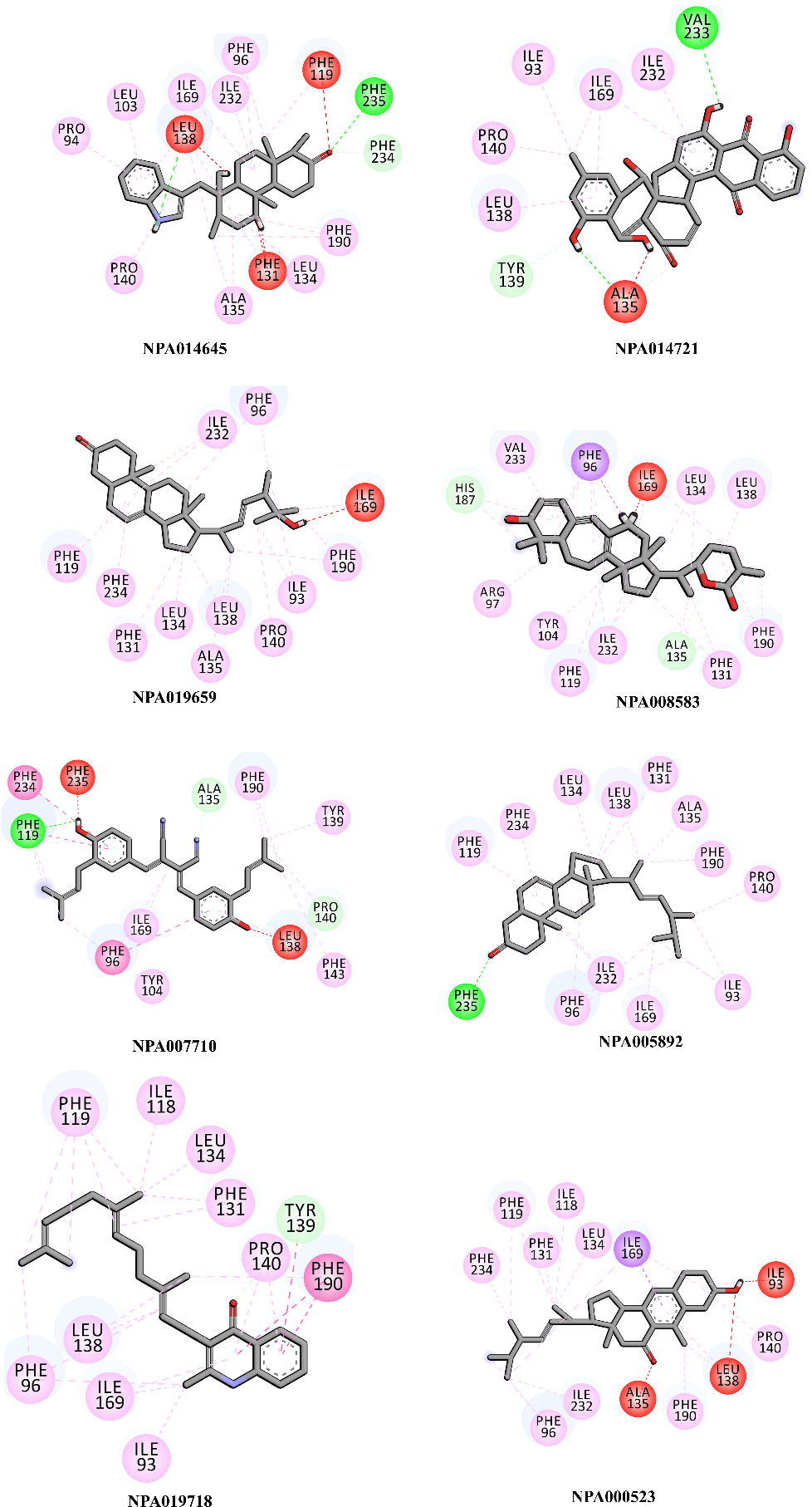


**Figure S1.** *Continued.*

*
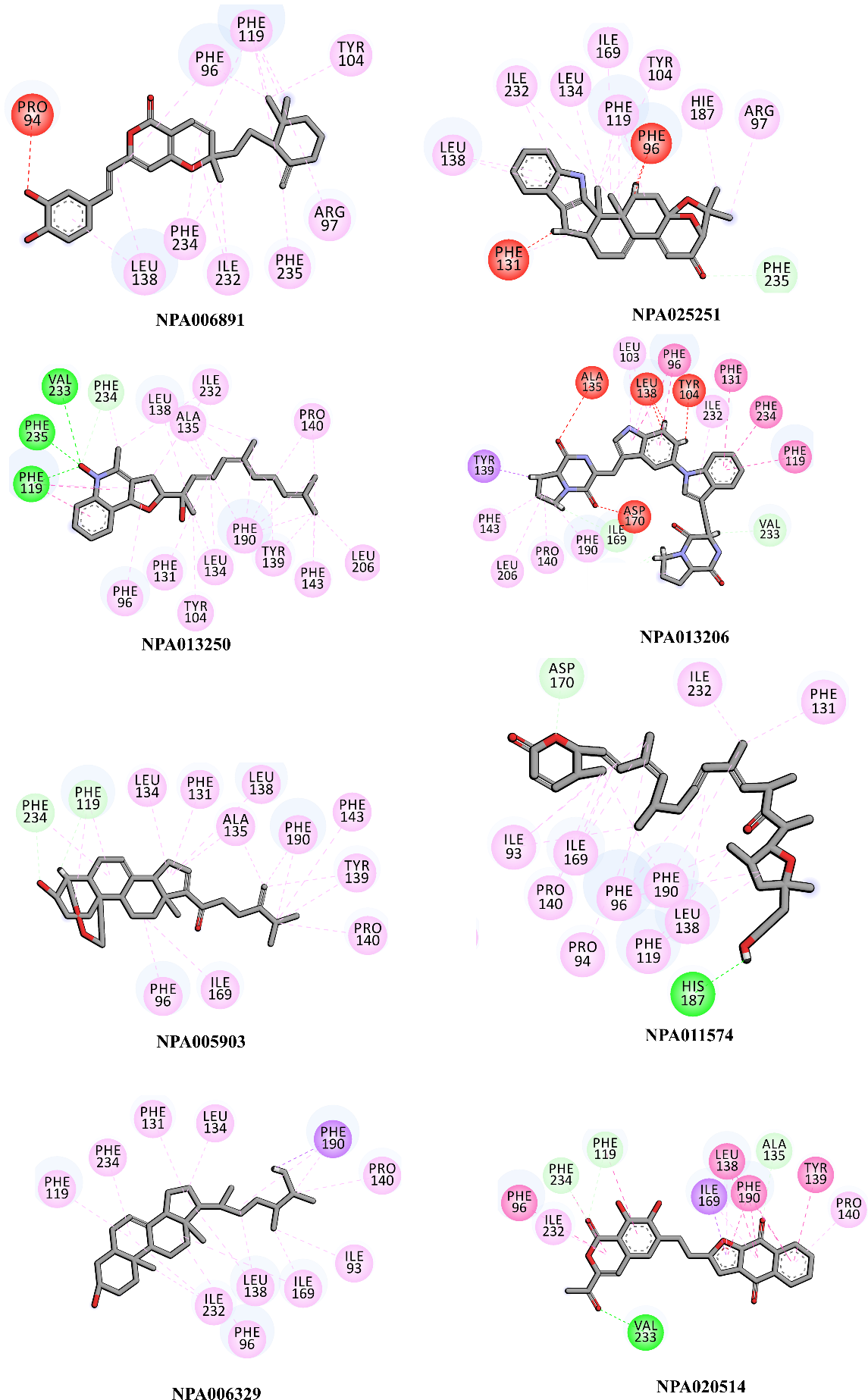
*

**Figure S1.** *Continued.*

**
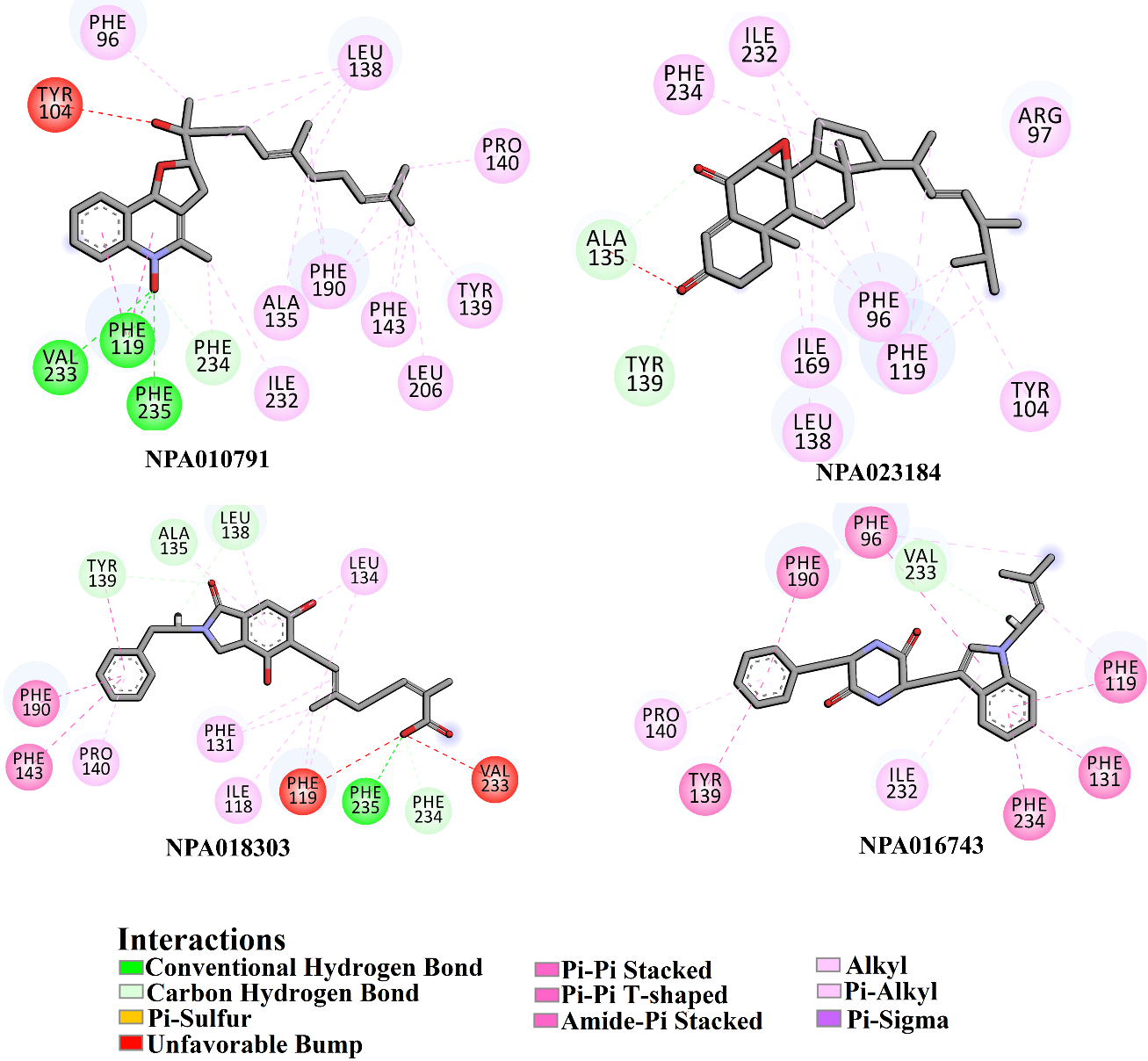
**

**Figure S1.** *Continued.*

**
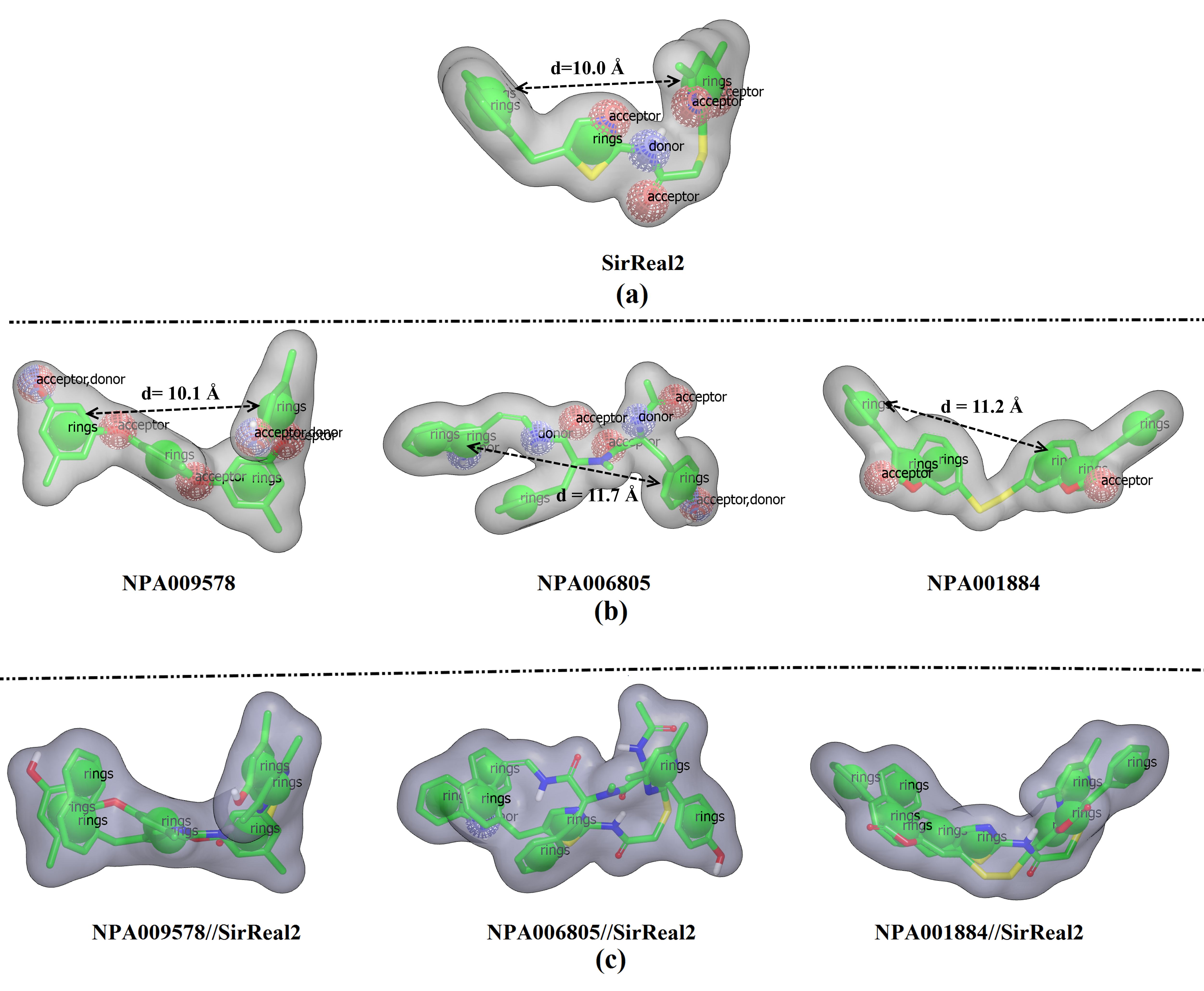
**

**Figure S2.** 3D pharmacophore models of **(**a) SirReal2, (b) identified NPAtlas compounds, and (c) superimposed pharmacophores of the identified NPAtlas compounds and SirReal2 (only aromatic rings were displayed for clarity). Distance between the essential aromatic rings in the pharmacophore model is given in Å. Pharmacophore models were developed by ROCS software (ROCS 3.2.2; OpenEye Scientific Software: Santa Fe, NM, USA, 2017).

**Table S1.** Estimated fast and expansive docking scores (in kcal/mol) and MM-GBSA binding energies (in kcal/mol) over 5 ns MD simulations for SirReal2 and the top potent NPAtlas compounds against SIRT2 protein.^a^

| No. | NPAtlas Code | **Docking Score (kcal/mol)** | | MM-GBSA Binding Energy (kcal/mol) | No. | NPAtlas Code | Docking Score (kcal/mol) | | **MM-GBSA Binding Energy (kcal/mol)** |
| --- | --- | --- | --- | --- | --- | --- | --- | --- | --- |
|  |  | Standard | Expensive |  |  |  | Standard | **Expensive** |  |
|  | SirReal2 | –11.8 | –12.0 | –46.4 | 34 | NPA025251 | –12.3 | –12.0 | –45.9 |
| 1 | NPA009578 | –13.3 | –13.4 | –60.0 | 35 | NPA013250 | –12.4 | –12.0 | –45.9 |
| 2 | NPA006805 | –13.1 | –13.2 | –57.3 | 36 | NPA013206 | –12.1 | –12.1 | –45.5 |
| 3 | NPA001884 | –13.0 | –13.1 | –55.6 | 37 | NPA005903 | –12.2 | –12.0 | –45.4 |
| 4 | NPA023712 | –13.0 | –12.9 | –53.8 | 38 | NPA011574 | –12.1 | –12.1 | –45.2 |
| 5 | NPA000470 | –12.9 | –12.9 | –53.8 | 39 | NPA006329 | –12.1 | –12.0 | –44.5 |
| 6 | NPA018719 | –12.7 | –12.8 | –53.4 | 40 | NPA020514 | –12.2 | –12.1 | –44.4 |
| 7 | NPA023225 | –12.6 | –12.5 | –53.3 | 41 | NPA010791 | –12.3 | –12.1 | –44.3 |
| 8 | NPA014478 | –12.5 | –12.4 | –52.1 | 42 | NPA023184 | –12.0 | –11.9 | --- |
| 9 | NPA001597 | –12.6 | –12.2 | –51.6 | 43 | NPA018303 | –12.1 | –11.9 | --- |
| 10 | NPA002757 | –12.3 | –12.1 | –51.6 | 44 | NPA016743 | –12.3 | –11.8 | --- |
| 11 | NPA011103 | –12.1 | –12.1 | –51.3 | 45 | NPA007879 | –12.5 | –11.8 | --- |
| 12 | NPA009593 | –12.0 | –12.1 | –51.1 | 46 | NPA009249 | –12.1 | –11.8 | --- |
| 13 | NPA010958 | –12.0 | –12.0 | –51.1 | 47 | NPA013441 | –12.1 | –11.7 | --- |
| 14 | NPA019551 | –12.8 | –12.1 | –51.0 | 48 | NPA000667 | –12.7 | –11.7 | --- |
| 15 | NPA005980 | –12.4 | –12.1 | –50.4 | 49 | NPA016249 | –12.1 | –11.7 | --- |
| 16 | NPA014305 | –12.1 | –12.1 | –50.3 | 50 | NPA017867 | –12.0 | –11.6 | --- |
| 17 | NPA011323 | –12.3 | –12.1 | –50.2 | 51 | NPA026452 | –12.4 | –11.5 | --- |
| 18 | NPA005296 | –12.1 | –12.1 | –50.2 | 52 | NPA005660 | –12.2 | –11.5 | --- |
| 19 | NPA020225 | –13.5 | –12.1 | –49.5 | 53 | NPA007365 | –12.2 | –11.5 | --- |
| 20 | NPA017119 | –12.1 | –12.1 | –48.9 | 54 | NPA025663 | –12.2 | –11.4 | --- |
| 21 | NPA024196 | –12.0 | –12.0 | –48.8 | 55 | NPA010664 | –12.1 | –11.4 | --- |
| 22 | NPA025117 | –12.4 | –12.1 | –48.7 | 56 | NPA011196 | –12.3 | –11.3 | --- |
| 23 | NPA018887 | –12.7 | –12.1 | –48.6 | 57 | NPA000040 | –13.0 | –11.3 | --- |
| 24 | NPA006062 | –12.2 | –12.0 | –48.4 | 58 | NPA019425 | –12.0 | –11.1 | --- |
| 25 | NPA014645 | –12.4 | –12.0 | –48.3 | 59 | NPA001450 | –12.3 | –11.1 | --- |
| 26 | NPA014721 | –12.9 | –12.0 | –47.6 | 60 | NPA002698 | –13.0 | –11.0 | --- |
| 27 | NPA019659 | –12.2 | –12.1 | –47.5 | 61 | NPA004041 | –12.0 | –11.0 | --- |
| 28 | NPA008583 | –12.0 | –12.0 | –47.0 | 62 | NPA009626 | –12.0 | –11.0 | --- |
| 29 | NPA007710 | –12.7 | –12.1 | –47.0 | 63 | NPA024841 | –12.0 | –11.0 | --- |
| 30 | NPA005892 | –12.7 | –12.7 | –46.9 | 64 | NPA027717 | –12.3 | –10.9 | --- |
| 31 | NPA019718 | –12.1 | –12.1 | –46.8 | 65 | NPA020778 | –13.2 | –10.8 | --- |
| 32 | NPA000523 | –12.1 | –12.1 | –46.2 | 66 | NPA010292 | –12.3 | –10.5 | --- |
| 33 | NPA006891 | –12.2 | –12.1 | –46.0 |  |  |  |  |  |

^a^Data sorted according to MM-GBSA binding energy over 5 ns MD simulations.

**Table S2.** Estimated fast and expansive docking scores (in kcal/mol), and MM-GBSA binding energies (in kcal/mol) over 5 and 50 ns MD simulations for SirReal2 and the top 31 potent NPAtlas compounds against SIRT2 protein.^a^

| No. | NPAtlas Code | **Docking Score (kcal/mol)** | | MM-GBSA Binding Energy (kcal/mol) | |
| --- | --- | --- | --- | --- | --- |
|  |  | **Fast** | **Expensive** | 5 ns | 50 ns |
|  | SirReal2 | –11.8 | –12.0 | –46.4 | –49.1 |
| 1 | NPA009578 | –13.3 | –13.4 | –60.0 | –60.9 |
| 2 | NPA006805 | –13.1 | –13.2 | –57.3 | –57.3 |
| 3 | NPA001884 | –13.0 | –13.1 | –55.6 | –54.6 |
| 4 | NPA023712 | –13.0 | –12.9 | –53.8 | –52.2 |
| 5 | NPA000470 | –12.9 | –12.9 | –53.8 | –52.0 |
| 6 | NPA018719 | –12.7 | –12.8 | –53.4 | –52.0 |
| 7 | NPA023225 | –12.6 | –12.5 | –53.3 | –51.7 |
| 8 | NPA014478 | –12.5 | –12.4 | –52.1 | –51.6 |
| 9 | NPA001597 | –12.6 | –12.2 | –51.6 | –51.2 |
| 10 | NPA018887 | –12.7 | –12.7 | –48.7 | –49.1 |
| 11 | NPA010958 | –12.0 | –12.0 | –53.3 | –49.1 |
| 12 | NPA005296 | –12.1 | –12.2 | –51.0 | –49.0 |
| 13 | NPA009593 | –12.0 | –12.1 | –53.4 | –49.0 |
| 14 | NPA017119 | –12.1 | –12.1 | –49.5 | –49.0 |
| 15 | NPA014645 | –12.4 | –12.4 | –48.3 | –49.0 |
| 16 | NPA002757 | –12.3 | –12.3 | –55.6 | –48.9 |
| 17 | NPA014305 | –12.1 | –12.1 | –51.6 | –48.9 |
| 18 | NPA011103 | –13.1 | –13.1 | –53.8 | –48.8 |
| 19 | NPA019659 | –12.2 | –12.2 | –47.5 | –48.7 |
| 20 | NPA008583 | –12.0 | –12.0 | –47.0 | –48.6 |
| 21 | NPA007710 | –12.7 | –12.7 | –47.0 | –48.3 |
| 22 | NPA019551 | –12.8 | –12.7 | –52.1 | –48.2 |
| 23 | NPA011323 | –12.3 | –12.2 | –51.3 | –48.2 |
| 24 | NPA025117 | –12.4 | –12.4 | –48.8 | –47.8 |
| 25 | NPA005892 | –12.7 | –12.7 | –46.9 | –47.6 |
| 26 | NPA006062 | –12.2 | –12.2 | –48.4 | –47.6 |
| 27 | NPA020225 | –13.5 | –13.4 | –50.2 | –47.6 |
| 28 | NPA019718 | –12.1 | –12.1 | –46.8 | –47.5 |
| 29 | NPA024196 | –12.0 | –12.0 | –48.9 | –47.4 |
| 30 | NPA005980 | –12.4 | –12.4 | –51.6 | –47.2 |
| 31 | NPA014721 | –12.9 | –12.9 | –47.6 | –46.6 |

^a^Data sorted according to MM-GBSA binding energy over 50 ns MD simulations.
